# The characteristics of EEG power spectra changes after ACL rupture

**DOI:** 10.1371/journal.pone.0170455

**Published:** 2017-02-09

**Authors:** Xin Miao, Hongshi Huang, Xiaoqing Hu, Dai Li, Yuanyuan Yu, Yingfang Ao

**Affiliations:** Institute of Sports Medicine, Peking University Third Hospital, Beijing, China; Mayo Clinic Minnesota, UNITED STATES

## Abstract

**Background:**

Reestablishing knee stability is the core of the treatment of ACL (Anterior Cruciate Ligament) injury. Some patients still have a feeling of instability of the knee after ACL injury treatment. This unstable feeling may be caused by central nervous system changes after ACL rupture.

**Methods:**

To identify the central changes after ACL rupture, EEG spectra were recorded to compare ACL patients and healthy controls when they were walking, jogging, and landing.

**Results:**

There was a significant increase in delta, theta, alpha and beta band power during walking, jogging and landing in ACL patients. We also found an asymmetry phenomenon of EEG only in the ACL patients, mainly in the frontal area and central-parietal area. The asymmetry of beta band power extended to the frontal and the central area during jogging and landing task.

**Conclusions:**

There were significant differences in EEG power spectra between the ACL patients and healthy people. ACL patients showed high EEG band power activities and an asymmetry phenomenon. EEG power changes were affected by movements, the asymmetry extended when performing more complicated movements.

## Introduction

Anterior cruciate ligament (ACL) rupture is one of the most common sport injuries. The high cost of ACL injury treatment, the long duration of recovery and the physical functional limitation after surgery are serious medical care problems. The ACL is one of the important structures of the knee joint in maintaining joint stability. After the ligament ruptures, the tibia is no longer restricted in its forward movement. This instability of the knee joint will cause meniscus injury, cartilage degeneration and other tissue damage, even osteoarthritis [1, 2]. Restoring knee joint stability is the key issue of ACL injury. At present, although arthroscopy of ACL reconstruction has satisfactory clinical effects [3, 4], some patients still feel unstable, even after surgery and physiotherapy [5–7].

Joint stability includes both static and dynamic stability. The static stability of the knee joint is mainly provided by the ligaments and capsule. However, the ACL not only plays a role as a mechanical connection but is also an important proprioceptor [8, 9]. When the knee joint bends, the ACL is stretched passively. This tension activates the proprioceptors. Then, these nerve impulses transfer to the central nervous system, causing proprioception, which provide muscle contraction and coordination for knee stability. This provides the dynamic stability of the knee joint [10]. Therefore, ACL rupture not only affects the static mechanical stability of the knee joint but can also lead to a loss of protective reflex and impairment of proprioception [11–14], which are important for the dynamic stability of the knee joint.

The dynamic instability of the knee joint after ACL rupture is a popular research topic. Most studies have focused on the peripheral region of the motor-sensory system, such as the EMG of the knee muscles. Only a few studies have paid attention to the change characteristics of the central nervous system. The central nervous system (CNS) is an important executive control center of the whole proprioceptive sensory pathway, which controls knee joint stability. Therefore, the CNS plays a crucial role in the knee’s functional stability.

An electroencephalogram (EEG) is recorded by electrodes. Brain electrical rhythms have a specific function and occur in specific areas. For example, Kristina Plattner investigated the relationship between the symptoms of EIMD (Exercise-induced-muscle-damage) and cortical beta activity during a submaximal biceps brachii movement. Beta-1 and -2 activity was increased in the frontal and parietal area in the experimental group. These data suggested that a change in β-1 and -2 activity was associated with integrating movement perception and proprioception post-EIMD [15]. Thus, EEG is an important source of data and window for studying changes in brain function. Compared with brain functional magnetic resonance imaging, EEG has a faster time resolution.

To date, there are only a few studies on EEG characteristics after ACL rupture. Baumeister found that the cortical activity showed higher frontal theta power during a force reproduction task with the reconstructed limb (F3 and Fz: p < 0.05) in the ACL group compared to the controls [16]. During a knee-angle reproduction task, brain activity demonstrated higher frontal theta power (F3, F4, F8) in both limbs of the ACL group vs. the controls, and a higher alpha-2 power was shown in the ACL-reconstructed limb compared with controls at parietal positions (P3, P4). These findings might indicate differences in focused attention with involvement of the anterior cingulate cortex (frontal theta) and sensory processing in the parietal somatosensory cortex (alpha-2) [17]. These studies showed modifications in EEG signals after ACL reconstruction. So far, it is not clear whether this is due to modifications in processing of proprioceptive sensory signals or the fear from hurt oneself again.

However, these studies did not measure EEG changes just after ACL rupture. These trials only recorded the EEG data after the ACL reconstruction operation, so their results were influenced by both the ACL sprain and the ACL reconstruction. Second, both trials only let the subjects do simple stability tasks, such as the joint position test (JPT) and force sense test. Real activities of daily living (ADL) such as walking or landing require more functional tasks. In addition, the studies used traditional wet electrode EEG recording devices, which limited the experimental design because they can’t nsynchronously record EEG during movement, and may bearing the patients extra weight, which could influence the motor task performance.

There are modifications of EEG signals after ACL reconstruction. The reasons of these modifications are not clear yet. One possibility is the lack of ACL leads to the changes of central nervous system. This study proposed the following hypothesis: compare with healthy subjects, the EEG band power of ACL rupture patients are different during movement. The purpose of this investigation was to measure the EEG readings of patients who have ACL rupture when performing various movements, compare the differences between the patients and healthy people, identify the EEG changes in the power spectrum caused by the ACL rupture.

## Methods

### Participants

In total, *n* = 31 male subjects were recruited for the study. Sixteen subjects (mean age 26.4 years, ranging from 20 to 32 years) were in the ACL rupture group, who only had ACL rupture injury (10 right side injuries and 6 left side injuries). Any other knee injuries, such as meniscus tear, cartilage injury and all somatic, neurological or psychiatric diseases, were excluded. All of these 16 subjects had pain or lower limbs functional impairment. And all of them had ACL reconstruction surgery after the EEG test. Fifteen healthy subjects (mean age 26.2 years, ranging from 20 to 30 years) were in the control group and were free of somatic, neurological or psychiatric diseases as well as any medication. Only male subjects were studied to avoid any confounding effects related to the menstrual cycle of the subjects. The lysholm score of ACL rupture group was significant different compare with the healthy control group, which showed the poor function and lower limbs impairment of the ACL rupture patients (Table 1). All participants were right-foot dominance and had no physical training experience. All participants refrained from physical exercise during the 24 hours before the test, and they had no physical or mental fatigue. This study was conducted after we obtained informed consent and Ethics Committee approval of Peking University Third Hospital. All of the participants provided their written informed consent to participate in this study.

**Table 1:** Subject Information.

	Number	Age (y)	Height (cm)	Body Weight (kg)	Duration of Injury (month)	Lysholm Score
ACL Rupture Group	16	26.4±6.31	176.6±8.63	80.9±14.11	8.6±7.22	81.7±13.09
Healthy Group	15	26.2±3.78	177.8±5.21	74.87±11.86	0	100

### EEG recording and quantification

EEG data were recorded by a Cognionics 32 channel amplifier with sampling at 1000 Hz. EEG signals were DC-recorded and band-pass-filtered from 0 to 100 Hz. The impedance of the electrodes was maintained under 5 *kΩ*. The EEG was recorded by Ag/AgCl electrodes mounted on a custom-made cap according to the extended 10–20 system. AFz served as the ground, and the left mastoid was referenced on-line. However, the EEG signal was re-referenced to the average of the bilateral mastoid electrodes offline.

Offline analysis was performed using the EMSE5.5.2 and Scan4.5 software, eliminating the blink artifacts and with low-pass filtering at 30 Hz. EEG epochs were segmented and baseline-corrected. Epochs more than 150 μ*V* were excluded as artifacts to ensure that each experimental condition had more than thirty effective trials. The band cut-offs were delta was <4 Hz, theta was between 4–7 Hz, alpha was between 8–13 Hz, and beta was between 13–30 Hz. We used the overall band power determined by integration of the power across the designated spectrum. The aspect of power analysis being in microvolts squared per octave (μV ^2^). All raw data were natural logarithm-transformed to produce data with a normal distribution. The ln-transformed data were extracted for statistical analysis using SPSS 18.0. The Greenhouse-Geisser correction was applied to the *p*-values.

### Movement task

The movement task included a walking test, jogging test and landing test. While wearing the EEG recording device, subjects walked 20 meters naturally. After the walking test, subjects rested 1 minute. Then, they started the jogging test, which consisted of jogging approximately 20 meters naturally without any pain. After 1 minute of rest, the subjects started the landing test. All participants jumped from a 25-cm-high step and landed with both legs naturally without any pain. Each participate had 7 movement cycles in every motor task. There was no trigger and the EEG date during the whole movement were recorded. 5 movement cycles were analyzed. The first and the last cycle were excluded.

## Results

### EEG power after ACL rupture during walking——Delta band

A mixed-model ANOVA with 3 locations (Fz vs. Cz vs. Pz) and 2 groups (ACL rupture group vs. healthy group) as the between-subject factor yielded an non-significant main effect of location (*F* (2, 58) = 0.662, *p* = 0.472, *η*^*2*^_*P*_ = 0.022), but there was a significant main effect of group (*F* (1, 29) = 116.064, *p* < 0.001, *η*^*2*^_*P*_ = 0.800). In addition, the interaction of location × group (*F* (2, 58) = 1.280, *p* = 0.279, *η*^*2*^_*P*_ = 0.042) was not significant. These results suggest that compared with the healthy group, the delta band power significantly increased in the ACL rupture group, and this increase was observed in all locations (Table 2, line 1–3, column 1–2).

**Table 2:** Delta, theta, alpha and beta band power at different locations during the walking test (μV^2^).

	ACL Rupture Group	Healthy Group
delta band		
Fz	14.28±2.44	7.26±1.85
Cz	13.80±2.19	7.38±1.74
Pz	14.60±1.88	7.25±1.91
F5	14.20±2.46	7.27±1.85
F6	16.91±3.01	7.96±1.31
C5	14.10±2.44	7.26±1.74
C6	14.40±1.87	7.27±1.69
P7	17.54±3.72	5.88±1.49
P8	15.20±2.63	5.89±1.66
theta band		
Fz	14.24±2.53	7.38±1.88
Cz	12.70±1.99	7.47±1.79
Pz	14.39±2.10	7.39±1.89
F5	14.19±2.50	7.40±1.86
F6	15.89±2.32	7.96±1.20
C5	14.10±2.48	7.38±1.76
C6	14.24±2.14	7.40±1.69
P7	16.54±3.69	5.65±1.66
P8	14.76±2.62	5.72±1.79
alpha band		
Fz	14.13±2.71	7.31±1.88
Cz	12.08±1.93	7.37±1.84
Pz	14.23±2.34	7.32±1.89
F5	14.07±2.68	7.34±1.85
F6	15.19±2.10	7.80±1.16
C5	13.98±2.65	7.30±1.78
C6	14.11±2.29	7.33±1.69
P7	15.61±3.44	5.29±1.84
P8	14.52±2.66	5.44±1.88
beta band		
Fz	13.62±2.65	7.14±1.87
Cz	11.26±2.05	7.19±1.87
Pz	13.63±2.58	7.16±1.87
F5	13.57±2.64	7.18±1.81
F6	14.23±2.02	7.52±1.20
C5	13.49±2.61	7.15±1.76
C6	13.59±2.30	7.16±1.69
P7	14.52±3.37	4.98±1.89
P8	13.84±2.64	5.14±1.92

A mixed-model ANOVA with 2 hemispheres (left vs. right) and 3 areas (F vs. C vs. P) as the within-subject factor and 2 groups (ACL rupture group vs. healthy group) as the between-subject factor yielded a significant main effect of area (*F* (2, 58) = 3.584, *p* = 0.049, *η*^*2*^_*P*_ = 0.110), but there was a non-significant main effect of hemisphere (*F* (1, 29) = 1.231, *p* = 0.276, *η*^*2*^_*P*_ = 0.041) and another significant main effect of group (*F* (1, 29) = 169.153, *p* < 0.001, *η*^*2*^_*P*_ = 0.854). In addition, the interactions of area × group (*F* (2, 58) = 17.090, *p* < 0.001, *η*^*2*^_*P*_ = 0.371) and area × hemisphere (*F* (2, 58) = 18.036, *p* < 0.001, *η*^*2*^_*P*_ = 0.383) were significant, but hemisphere × group was not significant (*F* (1, 29) = 0.002, *p* = 0.969, *η*^*2*^_*P*_ = 0.000). Moreover, the interaction of area × hemisphere × group was significant (*F* (2, 58) = 10.671, *p* = 0.001, *η*^*2*^_*P*_ = 0.269). We next compared the hemispheric differences of delta band power between the groups in the 3 areas. In the ACL rupture group, delta band power had hemispheric differences in the frontal area (*p* < 0.001) and central area (p = 0.029), in which the power in the left hemisphere was lower than the right hemisphere, and the parietal area (*p* < 0.001), in which the power in the left hemisphere was higher than the right hemisphere. In the healthy group, there were no hemispheric differences in any of the 3 areas (F, *p* = 0.328; C, *p* = 0.883; P, *p* = 0.979) (Table 2, line 4–9, column 1–2).

### EEG power after ACL rupture during walking——Theta band

A mixed-model ANOVA with 3 locations (Fz vs. Cz vs. Pz) and 2 groups (ACL rupture group vs. healthy group) as the between-subject factor yielded an significant main effect of location (*F* (2, 58) = 7.106, *p* = 0.010, *η*^*2*^_*P*_ = 0.197), and there was a significant main effect of group (*F* (1, 29) = 86.359, *p* <0.001, *η*^*2*^_*P*_ = 0.749). In addition, the interaction of location × group (*F* (2, 58) = 8.708, *p* = 0.005, *η*^*2*^_*P*_ = 0.231) was significant. These results suggest that compared with the healthy group, the theta band power significantly increased in the ACL rupture group. Therefore, we performed the simple effect analysis tests and found that the two groups had significant differences at Fz (*p* <0.001), Cz (*p* <0.001) and Pz (*p* <0.001)(Table 2, line 10–12, column 1–2).

A mixed-model ANOVA with 2 hemispheres (left vs. right) and 3 areas (F vs. C vs. P) as the within-subject factor and 2 groups (ACL rupture group vs. healthy group) as the between-subject factor yielded a non-significant main effect of area (*F* (2, 58) = 3.255, *p* = 0.067, *η*^*2*^_*P*_ = 0.101), hemisphere (*F* (1, 29) = 0.528, *p* = 0.473, *η*^*2*^_*P*_ = 0.018), but there was a significant main effect of group (*F* (1, 29) = 140.314, *p* < 0.001, *η*^*2*^_*P*_ = 0.829). In addition, the interactions of area × group (*F* (2, 58) = 16.869, *p* < 0.001, *η*^*2*^_*P*_ = 0.368), and area × hemisphere (*F* (2, 58) = 12.706, *p* < 0.001, *η*^*2*^_*P*_ = 0.305) were significant, but hemisphere × group (*F* (1, 29) = 0.374, *p* = 0.545, *η*^*2*^_*P*_ = 0.013) were not significant. The interaction of area × hemisphere ×group was also significant (*F* (2, 58) = 7.443, *p* = 0.004, *η*^*2*^_*P*_ = 0.204). Then, we compared the hemispheric differences of theta band power between the groups in the 3 areas. In the ACL rupture group, theta band power had hemispheric differences in the frontal area (*p* = 0.003), in which the power in the left hemisphere was lower than that in the right hemisphere, and the parietal area (*p* < 0.001), in which the power in the left hemisphere was higher than that in the right hemisphere. There were no hemispheric differences in the central area (*p* = 0.075). In the healthy group, there were no hemispheric differences in any of the 3 areas (F, *p* = 0.312; C, *p* = 0.782; P, *p* = 0.867) (Table 2, line 13–18, column 1–2).

### EEG power after ACL rupture during walking——Alpha band

A mixed-model ANOVA with 3 locations (Fz vs. Cz vs. Pz) and 2 groups (ACL rupture group vs. healthy group) as the between-subject factor yielded a significant main effect of location (F (2, 58) = 15.669, *p* <0.001, *η*^*2*^_*P*_ = 0.351) and group (F (1, 29) = 71.716, *p* < 0.001, *η*^*2*^_*P*_ = 0.712). In addition, the interaction of location × group (F (2, 58) = 17.466, *p* <0.001, *η*^*2*^_*P*_ = 0.376) was significant. These results suggest that compared with the healthy group, the alpha band power significantly increased in the ACL rupture group. Therefore, we performed the simple effect analysis tests and found that the two groups had significant differences at Fz (*p* < 0.001), Cz (*p* < 0.001) and Pz (*p* < 0.001) (Table 2, line 19–21, column 1–2).

A mixed-model ANOVA with 2 hemispheres (left vs. right) and 3 areas (F vs. C vs. P) as the within-subject factors and 2 groups (ACL rupture group vs. healthy group) as the between-subject factor yielded a significant main effect of area (*F* (2, 58) = 5.383, *p* = 0.018, *η*^*2*^_*P*_ = 0.157) and group (*F* (1, 29) = 120.853, *p* < 0.001, *η*^*2*^_*P*_ = 0.806), but non-significant of hemisphere (*F* (1, 29) = 0.889, *p* = 0.354, *η*^*2*^_*P*_ = 0.030),. In addition, the interactions of area × group (*F* (2, 58) = 18.239, *p* <0.001, *η*^*2*^_*P*_ = 0. 386) and area × hemisphere (*F* (2, 58) = 7.248, *p* = 0.005, *η*^*2*^_*P*_ = 0.200) were significant, but hemisphere × group (*F* (1, 29) = 0.342, *p* = 0.563, *η*^*2*^_*P*_ = 0.012) was not significant. The interaction of area × hemisphere × group was also significant (*F* (2, 58) = 4.311, *p* = 0.031, *η*^*2*^_*P*_ = 0.129). Then, we compared the hemispheric differences of alpha band power between the groups in the 3 areas. In the ACL rupture group, the alpha band power had hemispheric differences in the frontal area (*p* = 0.021), in which the power in the left hemisphere was lower than that in the right hemisphere, and the parietal area (*p* = 0.002), in which the power in the left hemisphere was higher than that in the right hemisphere. There were no hemispheric differences in the central area (*p* = 0.140). In the healthy group, there were no hemispheric differences in any of the 3 areas (F, *p* = 0.337; C, *p* = 0.785; P, *p* = 0.663) (Table 2, line 22–27, column 1–2).

### EEG power after ACL rupture during walking——Beta band

A mixed-model ANOVA with 3 locations (Fz vs. Cz vs. Pz) and 2 groups (ACL rupture group vs. healthy group) as the between-subject factor yielded a significant main effect of location (*F* (2, 58) = 24.218, *p* < 0.001, *η*^*2*^_*P*_ = 0.455) and group (*F* (1, 29) = 56.857, *p* < 0.001, *η*^*2*^_*P*_ = 0.662). In addition, the interaction of location × group (*F* (2, 58) = 25.666, *p* <0.001, *η*^*2*^_*P*_ = 0.470) was significant. These results suggest that compared with the healthy group, the beta band power significantly increased in the ACL rupture group. Therefore, we performed the simple effect analysis tests and found that the two groups had significant differences at Fz (*p* < 0.001), Cz (*p* < 0.001) and Pz (*p* < 0.001) (Table 2, line 28–30, column 1–2).

A mixed-model ANOVA with 2 hemispheres (left vs. right) and 3 areas (F vs. C vs. P) as the within-subject factors and 2 groups (ACL rupture group vs. healthy group) as the between-subject factor yielded a significant main effect of areas (*F* (2, 58) = 8.718, *p* = 0.004,*η*^*2*^_*P*_ = 0.231) and group (*F* (1, 29) = 56.875, *p* < 0.001, *η*^*2*^_*P*_ = 0.662), but non-significant of hemisphere (*F* (1, 29) = 0.636, *p* = 0.432, *η*^*2*^_*P*_ = 0.021). In addition, the interaction of area × group (*F* (2, 58) = 18.869, *p* < 0.001, *η*^*2*^_*P*_ = 0.394) was significant, but the interactions of area × hemisphere (*F* (2, 58) = 3.447, *p* = 0.053, *η*^*2*^_*P*_ = 0.106) and hemisphere × group (*F* (1, 29) = 0.318, *p* = 0.577, *η*^*2*^_*P*_ = 0.011) were not significant. The interaction of area × hemisphere ×group was also not significant (*F* (2, 58) = 2.246, *p* = 0.130, *η*^*2*^_*P*_ = 0.072). Then, we compared the hemispheric differences of beta band power between the groups in the 3 areas. In the ACL rupture group, beta band power had no hemispheric differences in the frontal area (*p* = 0.076), central area (*p* = 0.173) and parietal area (*p* = 0.058). In the healthy group, there were no hemispheric differences in any of the 3 areas (F, *p* = 0.372; C, *p* = 0.891; P, *p* = 0.657) (Table 2, line 31–36, column 1–2).

### EEG power after ACL rupture during jogging——Delta band

A mixed-model ANOVA with 3 locations (Fz vs. Cz vs. Pz) and 2 groups (ACL rupture group vs. healthy group) as the between-subject factor yielded a non-significant main effect of location (*F* (2, 58) = 0.609, *p* = 0.493, *η*^*2*^_*P*_ = 0.021), but there was a significant main effect of group (*F* (1, 29) = 171.593, *p* < 0.001, *η*^*2*^_*P*_ = 0.855). In addition, the interaction of location × group (*F* (2, 58) = 0.177, *p* = 0.759, *η*^*2*^_*P*_ = 0.006) was not significant. These results suggest that compared with the healthy group, the delta band power significantly increased in the ACL rupture group, and this increase was observed in all locations (Table 3, line 1–3, column 1–2).

**Table 3:** Delta, theta, alpha and beta band power at different locations during the jogging test (μV^2^).

	ACL Rupture Group	Healthy Group
delta band		
Fz	15.11±2.48	8.05±1.01
Cz	15.52±1.47	8.17±0.92
Pz	15.30±2.50	8.03±1.01
F5	15.17±2.61	8.05±1.01
F6	17.40±2.55	8.48±1.22
C5	14.89±2.63	8.00±1.00
C6	15.25±1.99	7.99±0.98
P7	17.80±3.95	6.57±1.32
P8	15.50±2.54	6.74±1.60
theta band		
Fz	15.05±2.44	8.25±0.96
Cz	14.67±1.74	8.35±0.84
Pz	15.16±2.45	8.24±0.96
F5	15.06±2.58	8.25±0.96
F6	16.72±2.13	8.58±1.05
C5	14.87±2.56	8.20±0.95
C6	15.14±2.04	8.18±0.93
P7	16.98±4.09	6.44±1.33
P8	15.30±2.72	6.66±1.63
alpha band		
Fz	15.00±2.43	8.13±1.09
Cz	13.66±1.94	8.15±0.93
Pz	15.09±2.44	8.12±1.09
F5	15.02±2.56	8.13±1.09
F6	16.19±1.83	8.36±1.06
C5	14.83±2.56	8.07±1.07
C6	15.06±2.07	8.06±1.05
P7	16.09±3.95	6.11±1.38
P8	15.13±2.68	6.33±1.66
beta band		
Fz	14.45±2.61	7.97±1.10

A mixed-model ANOVA with 2 hemispheres (left vs. right) and 3 areas (F vs. C vs. P) as the within-subject factors and 2 groups (ACL rupture group vs. healthy group) as the between-subject factor yielded a significant main effect of area (*F* (2, 58) = 3.827, *p* = 0.041, *η*^*2*^_*P*_ = 0.117), a non-significant main effect of hemisphere (*F* (1, 29) = 0.667, *p* = 0.421, *η*^*2*^_*P*_ = 0.022), and a significant main effect of group (*F* (1, 29) = 183.343, *p* < 0.001, *η*^*2*^_*P*_ = 0.863). In addition, the interactions of area × group (*F* (2, 58) = 13.441, *p* < 0.001, *η*^*2*^_*P*_ = 0.317) and area × hemisphere (*F* (2, 58) = 11.734, *p* = 0.001, *η*^*2*^_*P*_ = 0.288) was significant, but hemisphere × group was not significant (*F* (1, 29) = 0.080, *p* = 0.780, *η*^*2*^_*P*_ = 0.003). The interaction of area × hemisphere ×group was also significant (*F* (2, 58) = 9.601, *p* = 0.002, *η*^*2*^_*P*_ = 0.249). Then, we compared the hemispheric differences of delta band power between the groups in the 3 areas. In the ACL rupture group, delta band power had hemispheric differences in the frontal area (*p* = 0.001), in which the power in the left hemisphere was lower than that in the right hemisphere, and the parietal area (*p* < 0.001), in which the power in the left hemisphere was higher than that in the right hemisphere. There were no hemispheric differences in the central area (*p* = 0.071). In the healthy group, there were no hemispheric differences in any of the 3 areas (F, *p* = 0.506; C, *p* = 0.975; P, *p* = 0.750) (Table 3, line 4–9, column 1–2).

### EEG power after ACL rupture during jogging——Theta band

A mixed-model ANOVA with 3 locations (Fz vs. Cz vs. Pz) and 2 groups (ACL rupture group vs. healthy group) as the between-subject factor yielded a non-significant main effect of location (*F* (2, 58) = 0.347, *p* = 0.595, *η*^*2*^_*P*_ = 0.012), but there was a significant main effect of group (*F* (1, 29) = 144.551, *p* <0.001, *η*^*2*^_*P*_ = 0.833). In addition, the interaction of location × group (*F* (2, 58) = 0.849, *p* = 0.381, *η*^*2*^_*P*_ = 0.028) was not significant. These results suggest that compared with the healthy group, the theta band power significantly increased in the ACL rupture group, and this increase was found in all locations (Table 3, line 10–12, column 1–2).

A mixed-model ANOVA with 2 hemispheres (left vs. right) and 3 areas (F vs. C vs. P) as the within-subject factors and 2 groups (ACL rupture group vs. healthy group) as the between-subject factor yielded a significant main effect of area (*F* (2, 58) = 4.097, *p* = 0.039, *η*^*2*^_*P*_ = 0.124 and group (*F* (1, 29) = 160.674, *p* < 0.001, *η*^*2*^_*P*_ = 0.847), but non-significant of hemisphere (*F* (1, 29) = 0.657, *p* = 0.424, *η*^*2*^_*P*_ = 0.022). In addition, the interactions of area × group (*F* (2, 58) = 12.532, *p* <0.001, *η*^*2*^_*P*_ = 0.302) and area × hemisphere (*F* (2, 58) = 7.363, *p* = 0.006, *η*^*2*^_*P*_ = 0.202) were significant, but hemisphere × group (*F* (1, 29) = 0.079, *p* = 0.780, *η*^*2*^_*P*_ = 0.003) was not significant. The interaction of area × hemisphere ×group was significant (*F* (2, 58) = 6.744, *p* = 0.008, *η*^*2*^_*P*_ = 0.189). When we compared the hemispheric differences in theta band power between the groups in the 3 areas, in the ACL rupture group, theta band power had hemispheric differences in frontal area (*p* = 0.003), in which the power in the left hemisphere was lower than that in the right hemisphere, and the parietal area (*p* = 0.002), in which the power in the left hemisphere was higher than that in the right hemisphere. There were no hemispheric differences in the central area (*p* = 0.149). In the healthy group, there were no hemispheric differences in any of the 3 areas (F, *p* = 0.547; C, *p* = 0.936; P, *p* = 0.671) (Table 3, line 13–18, column 1–2).

### EEG power after ACL rupture during jogging——Alpha band

A mixed-model ANOVA with 3 locations (Fz vs. Cz vs. Pz) and 2 groups (ACL rupture group vs. healthy group) as the between-subject factor yielded a significant main effect of location (*F* (2, 58) = 6.775, *p* = 0.010, *η*^*2*^_*P*_ = 0.189) and group (*F* (1, 29) = 116.822, *p* < 0.001, *η*^*2*^_*P*_ = 0.801). In addition, the interaction of location × group (*F* (2, 58) = 7.398, *p* = 0.007, *η*^*2*^_*P*_ = 0.203) was significant. These results suggest that compared with the healthy group, the alpha band power significantly increased in the ACL rupture group. Therefore, we performed the simple effect analysis tests and found that the two groups had significant differences at Fz (*p* < 0.001), Cz (*p* < 0.001) and Pz (*p* < 0.001) (Table 3, line 19–21, column 1–2).

A mixed-model ANOVA with 2 hemispheres (left vs. right) and 3 areas (F vs. C vs. P) as the within-subject factors and 2 groups (ACL rupture group vs. healthy group) as the between-subject factor yielded a significant main effect of area (*F* (2, 58) = 6.640, *p* = 0.010, *η*^*2*^_*P*_ = 0.186) and group (*F* (1, 29) = 152.095, *p* < 0.001, *η*^*2*^_*P*_ = 0.840), but non-significant of hemisphere (*F* (1, 29) = 0.974, *p* = 0.338, *η*^*2*^_*P*_ = 0.032),. In addition, the interactions of area × group (*F* (2, 58) = 11.434, *p* = 0.001, *η*^*2*^_*P*_ = 0.283) and area × hemisphere (*F* (2, 58) = 4.114, *p* = 0.039, *η*^*2*^_*P*_ = 0.124) were significant, but hemisphere × group (*F* (1, 29) = 0.001, *p* = 0.992, *η*^*2*^_*P*_ = 0.001) was not significant. The interaction of area × hemisphere ×group was also significant (*F* (2, 58) = 4.133, *p* = 0.038, *η*^*2*^_*P*_ = 0.125). We compared the hemispheric differences of alpha band power between the groups in the 3 areas. In the ACL rupture group, alpha band power had hemispheric differences in the frontal area (*p* = 0.010), in which the power in the left hemisphere was lower than that in the right hemisphere, and the parietal area (*p* = 0.037), in which the power in the left hemisphere was higher than that in the right hemisphere. There were no hemispheric differences in the central area (*p* = 0.216). In the healthy group, there were no hemispheric differences in any of the 3 areas (F, *p* = 0.600; C, *p* = 0.935; P, *p* = 0.636) (Table 3, line 22–27, column 1–2).

### EEG power after ACL rupture during jogging——Beta band

A mixed-model ANOVA with 3 locations (Fz vs. Cz vs. Pz) and 2 groups (ACL rupture group vs. healthy group) as the between-subject factor yielded a significant main effect of location (*F* (2, 58) = 12.433, *p* = 0.001, *η*^*2*^_*P*_ = 0.300) and group (*F* (1, 29) = 87.324, *p* < 0.001, *η*^*2*^_*P*_ = 0.751). In addition, the interaction of location × group (*F* (2, 58) = 11.553, *p* = 0.001, *η*^*2*^_*P*_ = 0.285) was also significant. These results suggest that compared with the healthy group, the beta band power significantly increased in the ACL rupture group. Therefore, we performed the simple effect analysis tests and found that the two groups had significant differences at Fz (*p* < 0.001), Cz (*p* < 0.001) and Pz (*p* < 0.001) (Table 3, line 28–30, column 1–2).

A mixed-model ANOVA with 2 hemispheres (left vs. right) and 3 areas (F vs. C vs. P) as the within-subject factors and 2 groups (ACL rupture group vs. healthy group) as the between-subject factor yielded a significant main effect of area (F (2, 58) = 10.904, p = 0.001, η2P = 0.273) and group (F (1, 29) = 121.953, p < 0.001, η2P = 0.808), but non-significant of hemisphere (F (1, 29) = 1.317, p = 0.261, η2P = 0.043). In addition, the interaction of area × group (F (2, 58) = 12.709, p = 0.001, η2P = 0.305) was significant, but the interactions of area × hemisphere (F (2, 58) = 1.628, p = 0.213, η2P = 0.053) and hemisphere × group (F (1, 29) = 0.107, p = 0.746, η2P = 0.004) were not significant. The interaction of area × hemisphere ×group was also not significant (F (2, 58) = 2.198, p = 0.141, η2P = 0.070). Then, we compared the hemispheric differences of beta band power between the 2 groups in the 3 areas. In the ACL rupture group, beta band power had hemispheric differences in the frontal area (p = 0.020), in which the power in the left hemisphere was lower than that in the right hemisphere. There were no hemispheric differences in the central area (p = 0.234) and the parietal area (p = 0.288). In the healthy group, there were no hemispheric differences in any of the 3 areas (F, p = 0.690; C, p = 0.951; P, p = 0.607) (Table 3, line 31–36, column 1–2).

### EEG power after ACL rupture during landing——Delta band

A mixed-model ANOVA with 3 locations (Fz vs. Cz vs. Pz) and 2 groups (ACL rupture group vs. healthy group) as the between-subject factor yielded a non-significant main effect of location (*F* (2, 58) = 016, *p* = 0.910, *η*^*2*^_*P*_ = 0.001), but there was a significant main effect of group (*F* (1, 29) = 205.075, *p* < 0.001, *η*^*2*^_*P*_ = 0.876). In addition, the interaction of location × group (*F* (2, 58) = 0.245 *p* = 0.637, *η*^*2*^_*P*_ = 0.008) was not significant. These results suggest that compared with the healthy group, the delta band power significantly increased in the ACL rupture group, and this increase was observed in all locations (Table 4, line 1–3, column 1–2).

**Table 4:** Delta, theta, alpha and beta band power at different locations during the landing test (μV^2^).

	ACL Rupture Group	Healthy Group
delta band		
Fz	14.80±2.23	7.66±0.92
Cz	14.71±1.82	7.84±0.81
Pz	14.88±2.21	7.66±0.92
F5	14.65±2.46	7.67±0.92
F6	17.67±2.04	7.93±1.22
C5	14.67±2.35	7.61±0.92
C6	14.94±1.59	7.61±0.89
P7	17.60±3.21	6.48±1.35
P8	15.43±2.24	6.43±1.38
theta band		
Fz	14.75±2.21	7.89±0.92
Cz	13.3±1.60	7.93±0.79
Pz	14.71±2.30	7.88±0.92
F5	14.66±2.33	7.89±0.93
F6	16.61±1.33	8.07±1.09
C5	14.64±2.29	7.82±0.93
C6	14.85±1.69	7.80±0.92
P7	16.70±3.24	6.22±1.27
P8	15.03±2.50	6.22±1.35
alpha band		
Fz	14.59±2.40	7.83±1.06
Cz	12.28±1.74	7.83±0.97
Pz	14.48±2.59	7.83±1.05
F5	14.50±2.57	7.83±1.07
F6	15.97±1.38	7.99±1.14
C5	14.48±2.53	7.78±1.04
C6	14.69±1.83	7.77±1.03
P7	15.56±3.23	5.91±1.32
P8	14.87±2.52	5.89±1.42
beta band		
Fz	14.12±2.39	7.64±1.2
Cz	11.29±1.76	7.57±1.24
Pz	14±2.58	7.64±1.19
F5	13.95±2.83	7.64±1.2
F6	14.84±1.68	7.72±1.2
C5	13.91±2.79	7.59±1.17
C6	14.2±1.83	7.57±1.16
P7	14.5±3.23	5.52±1.29
P8	14.22±2.59	5.52±1.39

A mixed-model ANOVA with 2 hemispheres (left vs. right) and 3 areas (F vs. C vs. P) as the within-subject factors and 2 groups (ACL rupture group vs. healthy group) as the between-subject factor yielded a significant main effect of area (*F* (2, 58) = 4.095, *p* = 0.039, *η*^*2*^_*P*_ = 0.124), a non-significant main effect of hemisphere (*F* (1, 29) = 1.315, *p* = 0.261, *η*^*2*^_*P*_ = 0.043), and a significant main effect of group (*F* (1, 29) = 284.289, *p* < 0.001, *η*^*2*^_*P*_ = 0.907). In addition, the interactions of area × group (*F* (2, 58) = 13.874, *p* < 0.001, *η*^*2*^_*P*_ = 0.324) and area × hemisphere (*F* (2, 58) = 21.186, *p* < 0.001, *η*^*2*^_*P*_ = 0.422) were significant, but hemisphere × group was not significant (*F* (1, 29) = 0.609, *p* = 0.442, *η*^*2*^_*P*_ = 0.021). The interaction of area × hemisphere × group was also significant (*F* (2, 58) = 16.735, *p* < 0.001, *η*^*2*^_*P*_ = 0.366). When we compared the hemispheric differences of delta band power between the groups in the 3 areas, in the ACL rupture group, delta band power had hemispheric differences in the frontal area (*p* < 0.001), in which the power in the left hemisphere was lower than that in the right hemisphere, and the parietal area (*p* < 0.001), in which the power in the left hemisphere was higher than that in the right hemisphere. There were no hemispheric differences in the central area (*p* = 0.207). In the healthy group, there were no hemispheric differences in any of the 3 areas (F, *p* = 0.651; C, *p* = 0.996; P, *p* = 0.931) (Table 4, line 4–9, column 1–2).

### EEG power after ACL rupture during landing——Theta band

A mixed-model ANOVA with 3 locations (Fz vs. Cz vs. Pz) and 2 groups (ACL rupture group vs. healthy group) as the between-subject factor yielded a significant main effect of location (*F* (2, 58) = 5.516, *p* = 0.025, *η*^*2*^_*P*_ = 0.160), and there was also a significant main effect of group (*F* (1, 29) = 159.061, *p* < 0.001, *η*^*2*^_*P*_ = 0.846). In addition, the interaction of location × group (*F* (2, 58) = 6.306, *p* = 0.017, *η*^*2*^_*P*_ = 0.179) was significant. These results suggest that compared with the healthy group, the theta band power significantly increased in the ACL rupture group. Therefore, we performed the simple effect analysis tests and found that the two groups had significant differences at Fz (*p* < 0.001), Cz (*p* < 0.001) and Pz (*p* < 0.001) (Table 4, line 10–12, column 1–2).

A mixed-model ANOVA with 2 hemispheres (left vs. right) and 3 areas (F vs. C vs. P) as the within-subject factors and 2 groups (ACL rupture group vs. healthy group) as the between-subject factor yielded a significant main effect of area (*F* (2, 58) = 4.207, *p* = 0.043, *η*^*2*^_*P*_ = 0.127), a non-significant main effect of hemisphere (*F* (1, 29) = 0.486, *p* = 0.491, *η*^*2*^_*P*_ = 0.016), and a significant main effect of group (*F* (1, 29) = 236.803, *p* < 0.001, *η*^*2*^_*P*_ = 0.891). In addition, the interactions of area × group (*F* (2, 58) = 13.481, *p* = 0.001, *η*^*2*^_*P*_ = 0.317) and area × hemisphere (*F* (2, 58) = 17.117, *p* < 0.001, *η*^*2*^_*P*_ = 0.371) was significant, but hemisphere × group was not significant (*F* (1, 29) = 121, *p* = 0.731, *η*^*2*^_*P*_ = 0.004). The interaction of area × hemisphere × group was also significant (*F* (2, 58) = 14.039, *p* < 0.001, *η*^*2*^_*P*_ = 0.326). We next compared the hemispheric differences of theta band power between the groups in the 3 areas. In the ACL rupture group, theta band power had hemispheric differences in the frontal area (*p* < 0.001), in which the power in the left hemisphere was lower than that in the right hemisphere, and the parietal area (*p* < 0.001), in which the power in the left hemisphere was higher than that in the right hemisphere. There were no hemispheric differences in the central area (*p* = 0.183). In the healthy group, there were no hemispheric differences in any of the 3 areas (F, *p* = 0.625; C, *p* = 0.904; P, *p* = 0.997) (Table 4, line 13–18, column 1–2).

### EEG power after ACL rupture during landing——Alpha band

A mixed-model ANOVA with 3 locations (Fz vs. Cz vs. Pz) and 2 groups (ACL rupture group vs. healthy group) as the between-subject factor yielded a significant main effect of location (*F* (2, 58) = 15.149, *p* < 0.001, *η*^*2*^_*P*_ = 0.343) and group (*F* (1, 29) = 105.192, *p* < 0.001, *η*^*2*^_*P*_ = 0.784). In addition, the interaction of location × group (*F* (2, 58) = 15.149, *p* < 0.001, *η*^*2*^_*P*_ = 0.343) was significant. These results suggest that compared with the healthy group, the alpha band power significantly increased in the ACL rupture group. Therefore, we performed the simple effect analysis tests and found that the two groups had significant differences at Fz (*p* < 0.001), Cz (*p* < 0.001) and Pz (*p* < 0.001) (Table 4, line 19–21, column 1–2).

A mixed-model ANOVA with 2 hemispheres (left vs. right) and 3 areas (F vs. C vs. P) as the within-subject factors and 2 groups (ACL rupture group vs. healthy group) as the between-subject factor yielded a significant main effect of area (*F* (2, 58) = 8.096, *p* = 0.007, *η*^*2*^_*P*_ = 0.218), a non-significant main effect of hemisphere (*F* (1, 29) = 1.431, *p* = 0.241, *η*^*2*^_*P*_ = 0.047), and a significant main effect of group (*F* (1, 29) = 175.903, *p*< 0.001, *η*^*2*^_*P*_ = 0.858). In addition, the interactions of area × group (*F* (2, 58) = 13.433, *p* = 0.001, *η*^*2*^_*P*_ = 0.317) and area × hemisphere (*F* (2, 58) = 12.251, *p* < 0.001, *η*^*2*^_*P*_ = 0.297) were significant, but hemisphere × group was not significant (*F* (1, 29) = 888, *p* = 0.354, *η*^*2*^_*P*_ = 0.030). The interaction of area × hemisphere × group was also significant (*F* (2, 58) = 8.686, *p* = 0.002, *η*^*2*^_*P*_ = 0.230). We compared the hemispheric differences of alpha band power between the groups in the 3 areas. In the ACL rupture group, alpha band power had hemispheric differences in the frontal area (*p*< 0.001), in which the power in the left hemisphere was lower than that in the right hemisphere, and the parietal area (*p* = 0.048), in which the power in the left hemisphere was higher than that in the right hemisphere. There were no hemispheric differences in the central area (*p* = 0.230). In the healthy group, there were no hemispheric differences in any of the 3 areas (F, *p* = 0.649; C, *p* = 0.931; P, *p* = 0.949) (Table 4, line 22–27, column 1–2).

### EEG power after ACL rupture during landing——Beta band

A mixed-model ANOVA with 3 locations (Fz vs. Cz vs. Pz) and 2 groups (ACL rupture group vs. healthy group) as the between-subjects factor yielded a significant main effect of location (*F* (2, 58) = 26.247, *p* < 0.001, *η*^*2*^_*P*_ = 0.475) and group (*F* (1, 29) = 82.848, *p* < 0.001, *η*^*2*^_*P*_ = 0.741). In addition, the interaction of location × group (*F* (2, 58) = 23.858, *p*< 0.001, *η*^*2*^_*P*_ = 0.451) was significant. These results suggest that compared with the healthy group, the beta band power significantly increased in the ACL rupture group. Therefore, we performed the simple effect analysis tests and found that two groups had significant differences at Fz (*p* < 0.001), Cz (*p* < 0.001) and Pz (*p* < 0.001) (Table 4, line 28–30, column 1–2).

A mixed-model ANOVA with 2 hemispheres (left vs. right) and 3 areas (F vs. C vs. P) as the within-subject factors and 2 groups (ACL rupture group vs. healthy group) as the between-subject factor yielded a significant main effect of area (*F* (2, 58) = 11.024, *p* = 0.002, *η*^*2*^_*P*_ = 0.275), a non-significant main effect of hemisphere (*F* (1, 29) = 1.051, *p* = 0.314, *η*^*2*^_*P*_ = 0.035), and a significant main effect of groups, (*F* (1, 29) = 129.561, *p* < 0.001, *η*^*2*^_*P*_ = 0.817). In addition, the interaction of area × group (*F* (2, 58) = 13.826, *p* = 0.001, *η*^*2*^_*P*_ = 0.323) was significant, but hemisphere × group (*F* (1, 29) = 0.8517, *p* = 0.364, *η*^*2*^_*P*_ = 0.028) and area × hemisphere (*F* (2, 58) = 6.444, *p* = 0.012, *η*^*2*^_*P*_ = 0.182) were not significant. The interaction of area × hemisphere ×group was not significant (*F* (2, 58) = 4.890, *p* = 0.028, *η*^*2*^_*P*_ = 0.144). Then, we compared the hemispheric differences of beta band power between the groups in the 3 areas. In the ACL rupture group, beta band power had hemispheric differences in the frontal area (*p* = 0.001), in which the power in the left hemisphere was lower than that in the right hemisphere. here were no hemispheric differences in the central area (*p* = 0.218) and the parietal area (*p* = 0.333). In the healthy group, there were no hemispheric differences in any of the 3 areas (F, *p* = 0.778; C, *p* = 0.927; P, *p* = 0.986) (Table 4, line 31–36, column 1–2).

### Topographical maps of the spectra (delta, theta, alpha and beta band)

**Fig 1:**
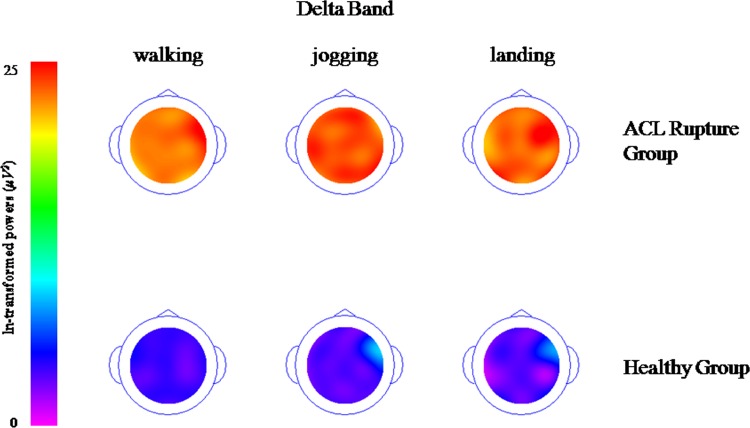
Topographical maps of the spectral delta band (It was ln-transformed in order to present the data clearly).

**Fig 2:**
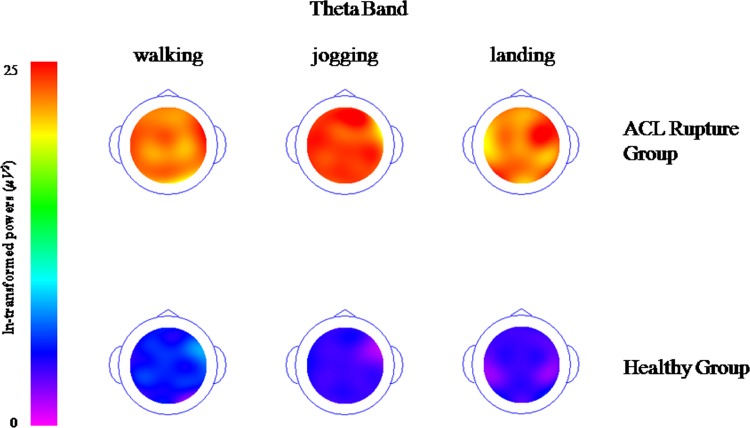
Topographical maps of the spectral theta band (It was ln-transformed in order to present the data clearly).

**Fig 3:**
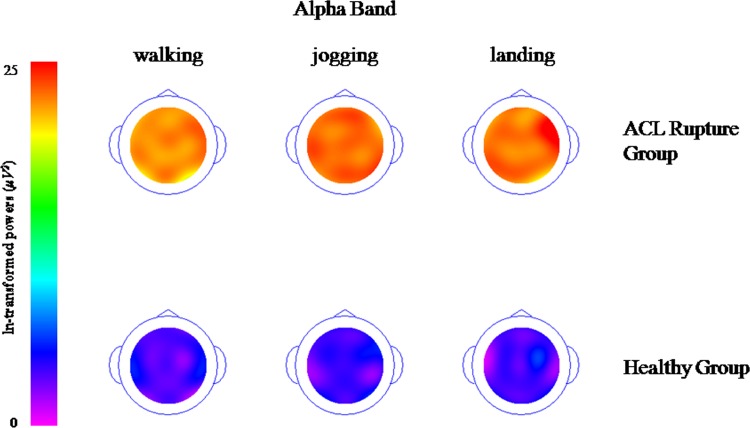
Topographical maps of the spectral alpha band (It was ln-transformed in order to present the data clearly).

**Fig 4:**
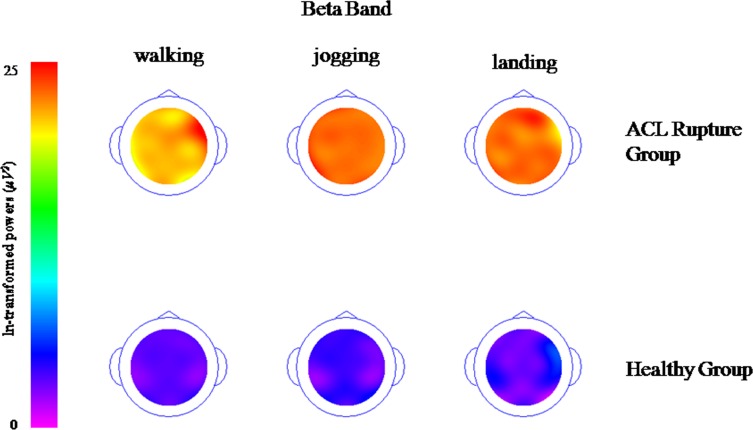
Topographical maps of the spectral beta band (It was ln-transformed in order to present the data clearly).

Topographical maps of the spectra show the descriptive demonstration of spectral Power in the different frequency bands, delta (Fig 1), theta (Fig 2), alpha (Fig 3) and beta band (Fig 4). These figures clearly demonstrate higher activations with different electrode locations in the ACL rupture patients compared to the healthy controls, which indicated by brighter colors (in this case orange) during walking, jogging and landing movement tasks.

## Discussion

### The characteristics of EEG power spectra of different frequencies after ACL rupture

To investigate the effects of ACL rupture on electrophysiology, we measured the EEG power change of different frequencies after ACL rupture. The delta, theta, alpha and beta band power of the subjects with ACL rupture was significantly higher than that of the healthy groups during walking, jogging and landing. Meanwhile, the power of different brain areas showed obvious asymmetries in the ACL rupture group. The power of the frontal lobe of the right hemisphere was higher than that of the left hemisphere, and the power of the parietal lobe of the left hemisphere was higher than that of the right hemisphere. The between-group distinction of the beta band was related to the motion styles. In particular, during the lower-limb stability task, which gave the ACL rupture group much difficulty, the asymmetry of beta band power in the brain extended to the frontal, which the power of the right hemisphere was higher than that of the left hemisphere, and the central area, which the power of the left hemisphere was higher than that of the right hemisphere.

The main finding of this study is that compared to the control healthy group, the delta, theta, alpha and beta band power of the ACL rupture group was significantly higher.

Baumeister [16,17] found that the brain activity demonstrates significantly higher frontal theta power (F3, F4, F8) in both limbs of the ACL group vs. the controls, and a significantly higher alpha-2 power was shown in ACL-reconstructed limbs compared with controls at parietal positions (P3, P4) during a knee-angle reproduction task. In another study, 9 patients after ACL reconstruction and 9 healthy controls were asked to reproduce 50% of their maximal voluntary isometric contraction (MVIC) reproduction. The cortical activity showed higher frontal theta power during the force reproduction task with the reconstructed limb (F3 and Fz: p < 0.05) of the ACL group compared to the controls. The results of these findings might indicate differences in focused attention with involvement of the anterior cingulate cortex (frontal theta) and sensory processing in the parietal somatosensory cortex (alpha-2). These findings are consistent with parts of the study, which might be related to the experimental conditions. First, the subjects of this study had ACL rupture but had not undergone reconstruction surgery. Second, this study investigated all areas of the anterior, central and posterior parts of the brain. Third, this study could better show the functional stability of the knee joint because the jogging, walking and landing tests were more closely related to people’s daily life activities. Fourth, all EEG data were recorded synchronously with the movement. Finally, this study found that the power of the delta band and theta band of the ACL rupture group became significantly higher during the three tests. The increase of delta and theta could be the interference noise for preventing proper execution. Also it could be an additional noise in the system due to lack of sensory input from the ACL proprioceptors. Another possibility is that the delta band and the theta band are sensitive during the movement.

We also found that in the ACL ruptured group alpha band power showed higher activity. Usually, increased alpha band (8–13 Hz) power shows the suppression of a process [18], selective cortical processing, and an idle cortex, and it is the parameter of the decline of the cortical activation [19]. In our study, during the walking, jogging and landing task, the ACL rupture patients needed more cognitive or attention, because of lack of afferent information. Some studies found that in cognitive or attention tasks, alpha band power increases in the areas that are not related to the tasks, which lead to the suppression of unrelated information [20–22]. Some studies have indicated that the alpha band reflects the cortical neuro-synchronization related to the excellent cognition-sport relationship of elite athletes, which means that high-level athletes have good concentration [23–25].

### The characteristics of EEG power spectra in different brain areas after ACL rupture

This study showed that the power of EEG signals in the frontal-parietal lobe became significantly stronger and was asymmetric during the tasks of jogging, walking and landing in the ACL rupture group.

The electric activities of the neurons of the pre-frontal lobe are involved in distinguishing signal meanings; spatial and non-spatial information maintenance and management; the preset, setoff, and adjustment of purposeful actions; and concentration or shift of attention. Almost all reports on pre-frontal damage describe distractibility after rupture of the pre-frontal lobe, which suggests the pre-frontal lobe is involved in attention and information storage. At present, it is believed that the pre-frontal lobe is important in information-abstracting, summarizing, and decision-making. The connection of the neuro-fibers of the parietal lobe is complex. Parietal lobules are involved in the fine motor movements of lateral limbs, and they can analyze and integrate stimuli from skin, arthrosis and tendons [26].

Above studies showed that the central motor area governs motor control, but the frontal and parietal lobes are involved in the feed-forwarding of signals, proprioception and fine motor movements. In our study, we also found the power of EEG signals in the frontal-parietal lobe was asymmetric in the ACL rupture group. This may be because ACL rupture influence the input information of proprioception and the signals of feed-forwarding process.

For the first time, this study detected specific effects on different brain areas caused by ACL rupture, especially the frontal and parietal lobes, and it offers some electrophysiological evidence for the cortical localization of lower-limb stability control.

### The characteristics of EEG power spectra during the different stability-requirement tasks

This study also found that in the different stability-requirement tasks, the asymmetry of beta band power in the brain extended to the frontal during jogging, which the power of the right hemisphere was higher than that of the left hemisphere, and the central area during landing task, which the power of the left hemisphere was higher than that of the right hemisphere.

Compared to walking, jogging and landing requires more stability. The most important function of the ACL is to restrict the tibia’s forward movement, especially when the knee is bending. When walking, the degree of knee joint bending is limited, and it does not challenge stability much. However, in landing, the ground reaction force can cause relative movements between tibia and femur, while the ACL is drawn tightly, which provides the force to draw and also acts as a mechanical sensor, sending information to the central cortex. Then, the central cortex can process this information to maintain the stability of landing. Compared to the healthy group, there was some loss of information to the central cortex in the ACL rupture group, so they needed more brain resources to maintain stability while landing. There have been very few EEG studies on lower-limb stability. Chang et al. investigated finger movements by resting-state fMRI and found that complex finger movement needed a larger cortical function area to adjust the movements, consistent with this study.

One limitation of our study is there were only male participants. The reason is to avoid the confounding effects related to the menstrual cycle of female subjects. However, female is at higher risk for both initial and secondary ACL injury. So one direction of the further studies needs to focus on female ACL rupture patients.

## Conclusion

There were significant differences in EEG power spectra between the ACL patients and healthy people. ACL patients showed high EEG band power activities and asymmetry. Additionally, EEG power changes were affected by movements, the asymmetry extended when performing more complicated movements.

## Supporting information

S1 File EEG data of ACL rupture group. (RAR)

S2 File EEG data of health control group. (RAR)
